# The voices not heard: thematic analysis of asylum seekers’ explanatory models of mental illness as elicited by the Cultural Formulation Interview

**DOI:** 10.1192/bjo.2024.866

**Published:** 2025-03-19

**Authors:** Lukas Claus, Mario Braakman, Meryam Schouler-Ocak, Laura Van de Vliet, Bernard Sabbe, Seline van den Ameele

**Affiliations:** 1 Collaborative Antwerp Psychiatric Research Institute (CAPRI), Universiteit Antwerpen (UA), Antwerp, Belgium; 2 Department of Psychiatry, Vrije Universiteit Brussel (VUB), Universitair Ziekenhuis Brussel (UZ Brussel), Brussels, Belgium; 3 Department of Criminal Law, Tilburg Law School, Tilburg University, Tilburg, The Netherlands; 4 Department of Psychiatry and Psychotherapy, Psychiatric University Clinic of Charité at St. Hedwig Hospital, Berlin, Germany; 5 POZAH Project (Asylum Seekers Mental Healthcare), St-Alexius Psychiatric Hospital, Grimbergen, Belgium; 6 Department of Psychiatry and Medical Psychology, Brugmann University Hospital, Brussels, Belgium

**Keywords:** Asylum seekers, cultural formulation interview, explanatory models, thematic analysis, mental health

## Abstract

**Background:**

Asylum seekers have difficulty gaining access to mental healthcare. Lack of understanding of asylum seekers’ mental illness explanatory models appears to be an important barrier. Gaining a better understanding of these explanatory models is crucial for ensuring the inclusion of asylum seekers in healthcare services. The Cultural Formulation Interview (CFI) might help to explore asylum seekers’ explanatory models of mental illness.

**Aims:**

To analyse asylum seekers’ explanatory models as elicited by the CFI.

**Methods:**

The CFI and its first supplementary module were carried out with asylum seekers with mental health problems. Transcriptions of the interviews underwent reflexive thematic analysis within a social constructivist framework.

**Results:**

In the analysis of 25 illness narratives, three major themes characterising asylum seekers’ explanatory models were identified: a burden of the past, a disenabling current reality, and a personal position and individual experience.

**Conclusions:**

The interplay among pre-, peri- and post-migration experiences, having a continuous impact on asylum seekers’ mental health, was highlighted by the themes ‘a burden of the past’, and ‘a disenabling current reality’. The theme ‘a personal position and individual experience’ revealed how the CFI enables self-determination in clinical encounters by embracing uncertainty and questioning the medicalisation of distress. The analysis characterises asylum seekers’ symptoms as a personal idiom of distress within socio-relational contexts. The CFI provides a clinically useful framework for exploring asylum seekers’ explanatory models and fostering dynamic understanding.

Mental illness is widespread among asylum seekers, with prevalence rates reaching 30% for post-traumatic stress disorder and depressive disorder.^1^ Among forcibly displaced people, traumatic experiences before and during migration interact with post-migration living difficulties (e.g. the precarious situation in camps, acculturation difficulties, lack of support) and significantly shape their distress.^2,3^ Asylum seekers face even greater vulnerability owing to factors such as insecure legal status, threat of detention or deportation, and family separation.^3,4^ Yet, their use of mental healthcare is low compared with their need, owing to a complex interplay of barriers.^5–7^ Among these barriers, divergent explanations of symptoms and treatment expectations between healthcare providers and patients undermine diagnostic accuracy. This may lead to inadequate treatment and negatively affect the therapeutic alliance. To overcome this different understanding, exploring patients’ explanatory models is suggested to improve mutual understanding and clinical outcomes.^8–10^

## Explanatory models

Kleinman introduced the term ‘explanatory model’ to describe ‘notions about an episode of sickness and its treatment that are employed by all those engaged in the clinical process’.^11^ This concerns the culturally influenced process of understanding one’s illness, assigning significance to symptoms, developing causal attributions, and articulating expectations on appropriate treatment and outcome.^8^ In formulating explanatory models, individuals use both personal and collective background knowledge and experience.^12^ Explanatory models are complex and dynamic constructs that can vary across clinical encounters, time and eco-social environment.^8,13,14^ First, the exploration of explanatory models helps to understand the significance of the illness and the patient’s wider beliefs. Paying attention to patients’ explanatory models is crucial for making clinical communication effective and ensuring treatment adherence.^8^ Second, explanatory models also involve the cognitive and social effects of mental illness, which have an impact on illness behaviour and concern the psychological and social response to illness. In this perspective, explanatory models do not solely attempt to explain illness experience but also have a fundamental role in the construction of psychiatric disorders and the response to them.^13^ Third, a migration experience can affect explanatory models. Familiarisation with cultural practices, beliefs and help-seeking strategies in the country of settlement, together with the acculturation process, may influence asylum seekers’ explanatory models.^12^ As a consequence, explanatory models are not simply cognitive schemas or isolated narratives but strategies for meaning-making that affect treatment-seeking behaviours and attitudes toward healthcare systems and practitioners.^13,15^ Therefore, understanding asylum seekers’ explanatory models is crucial for ensuring their engagement and inclusion in healthcare services.^15^

## Rationale for the current study

Despite the obvious need to gain an understanding of asylum seekers’ explanatory models, there is a lack of knowledge on how the experience of asylum-seeking influences explanatory models in the country of settlement.^12^ Various authors have explored explanatory models using a vignette and focus-group technique among refugees or first-generation migrants.^12,15,16^ Some studies have specifically focused on asylum seekers’ illness narratives and healthcare experiences without explicitly scoping explanatory models.^4,17,18^ Only a few European studies have evaluated explanatory models of mental health in asylum seekers, focusing on specific populations with a particular mental illness.^2,19^ To our knowledge, no study has evaluated asylum seekers’ explanatory models in a clinical context, without limitation to specific mental illnesses or countries of origin. Assessment tools such as the Explanatory Model Interview Catalogue and McGill Illness Narrative Interview are useful for research on explanatory models but are rather complex for clinical use.^8^ The DSM-5 Cultural Formulation Interview (CFI) could be a useful clinical tool for understanding a patient’s cultural context and explanatory models.^20^ The CFI consists of 16 questions designed to help clinicians elicit key cultural aspects of a patient’s clinical presentation. Explanatory models are specifically addressed in section 2 through four questions exploring the patient’s and their community’s causal attributions, perceived stressors and contextual support; these are further explored in the first supplementary module on explanatory models.^8^ Wallin and colleagues^21^ describe how CFI questions uncover information about patients’ explanations of distress. Although the use of cultural formulation to explore explanatory models has been suggested previously,^8^ there has been no more specific research on the use of the CFI to elicit information on explanatory models. The present study addresses this knowledge gap on asylum seekers’ explanatory models as elicited by the CFI. Gaining first-hand clinical knowledge of asylum seekers’ explanatory models is crucial for further initiatives on prevention, detection and care for asylum seekers with mental illness.^22^

## Methods

### Study design

This study is part of a larger research project on the use of CFI among asylum seekers. An open-access protocol paper detailing the design, procedure and research methodology of the complete project has been published previously.^22^ For the present work, an interpretative, qualitative study design was used to map out the main themes among explanatory models of asylum seekers that are identified by the CFI. This study design was based on the experiences and needs of stakeholders and refined through discussions with national and international experts in cultural psychiatry.^22^

### Participants and procedure

First-line healthcare workers and social workers of the asylum centres referred asylum seekers in need of a psychiatric assessment and willing to participate in the research project. Only asylum seekers who had mental illnesses and were capable of coherent verbal communication and written informed consent were included. Inclusion and intake procedures are described more widely in the protocol paper. This study focused on adult participants. All participants gave written informed consent, with the support of an interpreter if indicated. Following consent, 41 adult participants completed a clinical assessment in which the DSM-5 CFI and first supplementary CFI module (assessing explanatory models and illness prototypes) were administered. None of the participants dropped out. The first author (L.C.) conducted the interviews, after having received 2 days of training in conducting the CFI. The study team members are all clinical psychiatrists with experience in cultural psychiatry.

The authors assert that all procedures contributing to this work complied with the ethical standards of the relevant national and institutional committees on human experimentation and with the Helsinki Declaration of 1975, as revised in 2008. All procedures involving human participants were approved by the University of Antwerp’s ethics committee (BUN B3002022000005). Each participant received €15 per interview (maximum three interviews) as financial compensation. The interviews were audio-recorded and transcribed verbatim. Data were managed using REDCap, an electronic data capture tool.^23^ NVivo was used for the qualitative data analysis.^24^

### Data analysis

The CFI approach aims to jointly explore and co-construct meaningful narratives.^25^ As this study was based on the collaborative process through which the narrative of the explanatory model of the asylum seeker is brought forward – constructing meaning and knowledge through the interaction between the participant and the researcher–psychiatrist – it generally took an epistemological social constructivist stance.^26^ Principles of reflexive thematic analysis are applied to identify recurring themes related to asylum seekers’ explanatory models.^27,28^ Transcriptions of the core CFI and the first supplementary module were analysed. The analysis followed an inductive approach, without relying on any pre-existing theories, focusing on both semantic and latent levels of meaning. To maximise diversity, we considered both narrative richness and demographic characteristics when establishing the coding sequence. As data saturation is not a suitable concept in the context of reflexive thematic analysis, coding was continued as long as needed to obtain a sufficient depth of understanding.^29^ This was assumed to be the case after coding 25 narratives.

Two authors (L.C. and S.v.d.A.) coded the first four narratives, after which codes were compared to encourage reflexivity. Then, L.C. systematically coded the whole data-set, after which the process was revised iteratively, ascertaining that codes related to more than one data item.^27^ S.v.d.A. reviewed the coding process. L.C. built up a thematic framework, which was assessed by the co-authors to ensure internal coherence and distinguishability and iteratively adapted.^28^ L.C. further elaborated on the analysis for each theme and selected illustrative quotes. Randomly assigned identification numbers were linked to quotations to ensure anonymity.

## Results

**Fig. 1:**
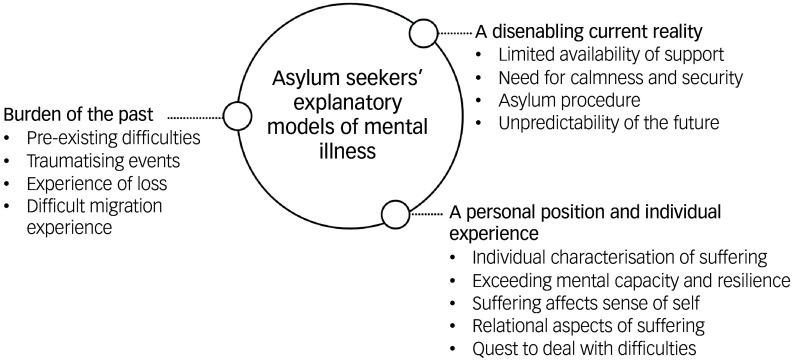
Overview of themes and subthemes.

We analysed illness narratives of 25 participants, of whom five (20%) identified as women. The participants’ ages varied between 18 and 29 years, with a median age of 26 years. Support from a certified interpreter was called upon for 21 (84%) participants. Table 1 summarises the interviewees’ characteristics. All of them were in a current asylum procedure in Belgium, living in a collective asylum reception centre. Four participants (16%) were temporarily residing in a hospital at the time of the interviews. Three overarching themes, each with several subthemes, were derived from the CFI-based illness narratives: ‘burden of the past’, ‘a disenabling current reality’, and ‘a personal position and individual experience’. These three main themes are summarised in Fig. 1 and elaborated in detail below.

**Table 1:** Participants’ characteristics

Participant	Age, years	Country of origin	Gender	Mother tongue	Interpreter
1	23	Somalia	Male	Somali	Yes
2	21	Afghanistan	Male	Pashto	Yes
3	27	Palestine	Male	Arabic	Yes
4	27	Burundi	Female	Kirundi	No
5	28	Syria	Male	Arabic	Yes
6	20	Cameroon	Male	French	No
7	22	Afghanistan	Male	Pashto	Yes
8	22	Burundi	Female	Kirundi	Yes
9	19	Niger	Male	Djerma	Yes
10	18	Senegal	Male	Wolof	Yes
11	21	Iraq	Male	Arabic	Yes
12	22	Afghanistan	Male	Pashto	Yes
13	28	Palestine	Male	Arabic	Yes
14	26	Georgia	Female	Georgian	Yes
15	28	Ghana	Male	Tui	No
16	28	Morocco	Male	Arabic	Yes
17	20	Afghanistan	Male	Pashto	Yes
18	30	Macedonia	Female	Serbian	Yes
19	21	Egypt	Male	Arabic	Yes
20	28	Eritrea	Male	Tigrinya	Yes
21	28	Iraq	Male	Arabic	Yes
22	27	Afghanistan	Male	Pashto	Yes
23	18	Palestine	Male	Arabic	Yes
24	29	Russia	Female	Russian	Yes
25	26	Burundi	Male	French	No

### Burden of the past

The theme ‘burden of the past’ reflected the enduring impact of previous experiences on participants’ current mental distress. Pre-existing difficulties formed a first subtheme. This included a history of mental health difficulties already present before departure, sometimes since early childhood. This subtheme also related to pre-migration experiences of political or familial insecurity. Some participants mentioned the difficult geopolitical situation in their country of origin.

‘When I was 6 years old, I had to flee with my father to Syria. I always had to flee from one country to another. From one city to another… I also had problems with Saddam Hussein’s government.’ (participant 11, male, Iraq)

Traumatising events formed a second major subtheme referring to a burdensome past. The traumatic events encompassed experiences of violence, abuse, inhumane treatment and life-threatening situations. Participants reported the long-lasting and ongoing impact of life-threatening experiences and how they led to anxiety regarding their own physical and psychological integrity.

‘They beat us, stopped us, took money from us. They undressed us, put us on an inflatable boat and sent us back. They put a hole in the inflatable boat so we could not go any further.’ (participant 23, male, Palestine)

The experience of loss further contributed to the burden of the past. Whether material loss, painful separation from family members or even the death of loved ones, the impact of these losses was profound. Distance from family caused sorrow, as did yearning for their presence. For some, the loss extended beyond their family to include their homeland and culture.

‘The Taliban came into my house in the middle of the night, and they killed my parents while they were looking for me. I was not at home. That is the most painful experience in my life.’ (participant 22, male, Afghanistan)

Participating asylum seekers’ difficult migration experience represents the last subtheme within the burden of the past. They report that they had no alternative but to leave their country. Some mention that their mental health only got worse since they left their country.

‘When I fled the country, they threatened me with guns. There are several causes. First, I had to go through a forest. Then they forced me with a gun to get into the boat… That stress has only increased since I left my homeland.’ (participant 11, male, Iraq)

In summary, the theme ‘burden of the past’ underscores the complex interplay among past and traumatic experiences, losses and the migration experience. All these aspects contributed to the enduring impact of the past on participants’ current mental health experience.

### A disenabling current reality

A second theme encompasses how the challenging current reality is experienced as disenabling by asylum seekers.

‘The situation I live in now has broken me mentally.’ (participant 11, male, Iraq)

The limited availability of support in the reception centres compounded the first subtheme. In the first place, participants struggled with a pervasive feeling of their needs being overlooked. Their need to be seen and heard in their suffering often went unaddressed, and this was compounded by experiences of disrespectful treatment. Some also perceived a lack of effectiveness in conventional care.

‘I see a lot of hypocrisy, no one cares about you; people ask: “Hi how are you,” but those people are not concerned about you, as if it is just a task.’ (participant 5, male, Syria)

Second, participants found that they could only rely on medical or social assistance owing to a loss of control; they could only share their suffering with caregivers and perceived medical support as essential for resolving their issues.

‘Look, just talking is not enough. You are a doctor; you know best what is good and what is not good for me. I think I need medication.’ (participant 22, male, Afghanistan)

Last, despite the challenges, receiving help elicited feelings of gratitude and fostered trust in caregivers. Being capable of engaging in dialogue with caregivers was perceived as helpful.

‘I respect someone who wants to listen to me and help me… I want to thank you for that.’ (participant 19, male, Egypt)

A second subtheme within the disenabling current reality related to asylum seekers’ need for calmness and security. Basic needs formed a cornerstone of this subtheme, with asylum seekers yearning for fundamental provisions such as peace, security and stability. The need for stability was related to the constant fear of ending up in a precarious situation. Struggling with the living conditions in a reception centre further intensified participants’ suffering, owing to overcrowding, strange food, a lack of privacy, etc.

‘It’s a source of stress here… I get nervous and sick because of the people who are here.’ (participant 11, male, Iraq)

The impact of an ongoing asylum procedure constituted the third aspect of the disenabling current reality. Participating asylum seekers reported fear of being deported back to their home countries. They also had trouble coping with the length of the asylum procedure and the lack of perspective. Moreover, they experienced significant distress because of the asylum procedure itself and saw it as a major source of their suffering. Participants indicated that recognition as a refugee was crucial to improvements in mental health.

‘I was rejected in Sweden. I fear I will suffer the same fate as in Sweden, which makes me very worried. Applying for asylum is not my ultimate goal. It is a matter of life and death.’ (participant 13, male, Palestine)

A last subtheme was characterised by the unpredictability of the future. A lack of perspective occupied asylum seekers’ thoughts, causing worry about what lay ahead. Their sense of responsibility towards their families intensified their worries. Unfulfilled expectations of a better life in Belgium increased their distress.

‘But if I feel this bad now, I’m not so sure how it’s going to be in the future… I thought coming here in search of a better life, coming to Europe, that I would find peace…’ (participant 7, male, Afghanistan)

They often hoped for a favourable evolution, whether in the form of a promising life in Belgium or any positive change. Nevertheless, this hope was sometimes overshadowed by feelings of hopelessness, exacerbated by the unpredictability of the asylum procedure. Some participants mentioned that this hopelessness provoked suicidal thoughts, underscoring the immense psychological toll of navigating an uncertain future within the asylum-seeking process.

‘The answer I get in my head is to kill myself. My life has expired. I have nothing left to do here.’ (participant 13, male, Palestine)

### A personal position and individual experience

The theme of ‘personal position and individual experience’ involved participating asylum seekers’ individual experiences of distress that exceeded their mental capacities, as well as affecting their sense of self, relational aspects and help-seeking strategies.

The first subtheme encompassed asylum seekers’ individual characterisation of their problem in the here and now. First, although participants regarded their current functioning and behaviour as a problem, they characterised this distress as a normal reaction to their current life circumstances. Symptoms such as hearing voices or seeing deceased ancestors were perceived as functional impairments, related to the current context rather than psychopathology.

‘But if you keep everything (problems, emotions) inside… there comes a time when I will explode… and because of the anger, then I can’t control myself and do many things, and after it I feel disappointed, I ask myself “why did I do that?”’ (participant 19, male, Egypt)

Second, many participants felt that they lacked the strength to keep going; they felt exhausted and even like they had had enough of life.

‘I feel I have died, I am just a corpse walking, among people. A living dead.’ (participant 13, male, Palestine)

Third, struggles with vivid memories were frequently reported. Some participants had vivid dreams or nightmares and found themselves ruminating. However, they found that with time, these memories started to fade. Some also mentioned that the distance from their country of origin helped them to cope with these memories.

‘When I lost my 15-year-old brother in Iran, that was the hardest. In Greece, and Turkey I got regular nightmares, that was about my little brother… They are horrible nightmares, in my nightmares I see cops killing my brother; when I wake up, I feel empty, and that makes me feel bad for several days in a row.’ (participant 12, male, Afghanistan)

In addition, they reported difficulties in their relationships with others, experiencing feelings of anger and distrust.

‘Loss of trust in the others makes it very bad, I no longer have trust because of what I went through.’ (participant 5, male, Syria)

Last, some participating asylum seekers outlined their difficulties as a physical problem. Bodily symptoms such as sudden awakenings, insomnia, loss of appetite, shortness of breath, palpitations or overloaded brains were mentioned. Physical symptoms were understood as an expression of the actual distress. Several expressed concerns about stress negatively affecting their physical health.

‘My fear is mainly at night when I am sleeping. Then my bed is completely soaked with sweat… then I can’t breathe… Sometimes when I lose control, it seems as if someone has beaten up my brain.’ (participant 1, male, Somalia)

A second subtheme concerned the experience of exceeding one’s own mental capacity and resilience.

First, participants frequently reported a loss of control regarding their complaints. This could be triggered by seemingly innocuous encounters or situations that elevated arousal levels. Interpersonal interactions in particular could provoke loss of control. Simply engaging in a conversation could be triggering.

‘She likes to listen to me, I’m the one who doesn’t like to talk about those things; […] if I talk about it too much, my situation is going to get worse. Talking about it makes me even more nervous.’ (participant 59, male, Iraq)

Although severe distress was mentioned as the cause of the loss of control, its manifestation significantly exacerbated participants’ suffering, as it rendered them unable to effectively manage their behaviour. Loss of control also provoked fears of becoming crazy or getting sick, or of the recurrence of traumatic experiences. Unable to predict the evolution of their symptoms or to regulate them, asylum seekers reported a persistent deterioration of their mental health. Some reported that this loss of control also engendered a fear of committing suicide.

‘Sometimes I don’t realise how serious it is and then I think I am going crazy… The anger then begins to creep into me and then I lose control of myself.’ (participant 1, male, Somalia)

Second, participating asylum seekers experienced powerlessness in facing their problems. They felt unable to address their mental illness alone and to communicate about their struggles. The inability to change anything about the broader situation in which they felt stuck was also repeatedly reported. Different participants tried to flee this sense of powerlessness. Some tried alcohol, whereas others felt ambivalent about using drugs. Others regained a sense of power by thinking of suicide or engaging in self-injurious behaviour.

‘I don’t want to be here so deeply. I can’t change anything because my life is my life… I feel everything I’m trying is not working. Sometimes I think it is not so bad if I died.’ (participant 14, female, Georgia)

The third subtheme encompassed how suffering affected asylum seekers’ sense of self. The first level of an affected sense of self encompassed how participants’ suffering influenced their self-perception. Their suffering was characterised by a range of distressing symptoms that had an impact on their entire being, such as fear, shame, guilt, not feeling free, mood swings and sadness. They often identified themselves with their suffering, which made them feel crazy and worthless.

‘What comes to my mind the most is the death of my father; I think about that daily… The problem I have is the guilt. How will I stop feeling guilty about the death of my father?’ (participant 3, male, Palestine)

A second level of impact on the sense of self was the experience of being affected as a human. Encounters with discrimination or racism were particularly painful and could trigger a profound sense of injustice. Participants also often reported being targeted for who they are, which extended to feeling as if they were being denied the fundamental right to exist. This perception seemed particularly pronounced among individuals who were not heterosexual or belonged to ethnic minorities.

‘I have no rights, am not independent… Isn’t that unjust that I experience such a thing? […] I went through a lot, suffered before… because we belonged to Kurdish Alawites, which made situation worse… Here you pretend respect for human rights. Is that just a slogan? Advertising? Or reality? […] You enjoy life, and we are not allowed to? That is not human, that is not humanity.’ (participant 5, male, Syria)

The last aspect of an affected sense of self was the confrontation with a stranger within. Suffering was a novel, unfamiliar experience and difficult to comprehend. The negative effect was perceived as intrusive and ego-dystonic, with participants describing feeling like they were no longer who they were.

‘In the beginning, my anger started creeping into me very smoothly, without me noticing it myself.’ (participant 1, male, Somalia)

They also experienced a fragmented sense of self that defied understanding. Some encountered a split between their inner and outer world and struggled to reconcile both realities. They may have struggled to bridge the gap between reality and a spiritual or imaginary world and felt disconnected from the present moment.

‘I was asking myself: ‘Why me, am I stupid?’ […] I don’t have any idea as I don’t even understand myself… My inside and outside are totally different… Now we are sitting together, and I can talk, but there is someone inside me reacting like I am crazy.’ (participant 4, female, Burundi)

Confronted with this alien part within themselves, participating asylum seekers resisted viewing themselves as sick, yearning to retrieve their former selves. They often avoided seeking help to evade being labelled as mentally ill.

‘We do everything not to be seen. They say if you go for a psychiatrist, you are mad.’ (participant 14, female, Georgia)

The fourth subtheme within the asylum seeker’s individual experience encompassed the relational aspects of their suffering. Participants often felt alone, unsupported and not understood. As their suffering often remained unnoticed, they felt lonely, believed they must solve their problems by themselves and struggled to connect with fellow asylum seekers who might share similar difficulties. Furthermore, they felt hesitant about sharing their experiences, as they perceived it to be unnecessary to burden others or considered their problems too personal to disclose.

‘Because there was no one I could talk with, no one was understanding me… I’m social right now but not that social to tell my full life because it’s not easy. Maybe there are people who are like me, but I can’t tell… To change, I have to do it myself… Many people want to talk to me, but inside of me it is different; [my inside wants] to be locked.’ (participant 4, female, Burundi)

Nevertheless, participants also mentioned sources of external reassurance. Other people, especially fellow asylum seekers, were perceived as reassuring when they expressed concern. Some also mentioned drawing strength in prayer and faith.

‘I pray five times a day. Begging Allah to change my situation… Of course, I have been going to the psychologist for a long time, and he has reassured me… [About a fellow asylum seeker:] He reassures me, he says it will be alright, and the doctor says the same… I just feel at ease with him, I can forget things when I’m with him.’ (participant 12, male, Afghanistan)

Last, they often expressed a profound need for connection with others and aspired to meaningful engagement in daily life through education, employment and social integration.

‘I very much want to get my own life back. Going to school, going to work, being outside with people.’ (participant 22, male, Afghanistan)

The last subtheme of the individual experience of distress concerned participants’ quest to deal with their difficulties. Many felt helpless and stuck as they could not identify anything helpful.

‘I don’t know how to deal with the situation I’m in. I would like to hear from someone else on how to deal with this. I have no idea.’ (participant 17, male, Afghanistan)

They described operating in survival mode, tackling challenges day by day while avoiding dwelling on the past or future. They attempted to dismiss negative thoughts and rather accept their situation. Many had been advised to seek distraction and keep busy, for example, in work, music, writing or sports or by going out in nature.

‘People say the past should be your past. But there are things of the past that age with you… even though I try to forget it doesn’t work… I try to think of positive things. One is that I still go to work even though I am tired, even though I feel bad. The other is exercising. Everything that happens to me is my fate, there is nothing I can do about it.’ (participant 16, male, Morocco)

The feeling of ‘not wanting to remember’ was repeatedly. Participants indicated that they avoided ruminating about the past, present or future. They desired to be alone and not be confronted. Concealment was also often advised by others. They linked their inability to conceal with increasing distress. However, concealment of negative feelings and avoidance of rumination were also reported to demand considerable energy.

‘I like to listen good music … to write my past; I try to like a normal person forget everything, but I don’t manage. I was trying to delete some pictures, to erase the memory, I want to be lucky… Because right now I need to accept things as they are… I fight the bad things; I put a lot of energy. It is working somehow… It is a survival strategy.’ (participant 4, female, Burundi)

Despite their suffering, some participating asylum seekers aimed for personal growth, focusing on positive aspects of life and striving to adapt to new circumstances. They viewed setbacks as opportunities to develop resilience.

‘We sometimes say that you become strong when you go through difficulties.’ (participant 25, male, Burundi)

## Discussion

This study provides first-hand insights into participating asylum seekers’ explanatory models of mental illness as elicited by the CFI and its first supplementary module. We identified three main themes shaping asylum seekers’ explanatory models: ‘burden of the past’, ‘a disenabling current reality’, and ‘a personal position and individual experience’. The first two themes are strongly linked with the previously described interaction between pre- and peri-migration traumatic experiences and post-migration living difficulties.^2^ Most salient explanations from the past concern not only trauma but also experiences of grief, loss and insecurity. Participants repeatedly mentioned the profound impact of these experiences. Previous research has confirmed these types of traumatic experience to be related to common mental disorders in asylum seekers.^30^ The theme ‘a disenabling current reality’ illustrates, in line with prior research, how post-migration living difficulties further explain asylum seekers’ distress.^2,30^ Earlier work confirms the limited availability of support at reception centres, with asylum seekers’ needs being overlooked.^18^ In many places, care ethics principles are suspended for migrants and refugees, leading to numerous barriers.^31^ Asylum seekers’ perception of relying solely on medical or social assistance owing to a loss of control illustrates the risk of medicalising their distress, rendering them vulnerable and passive and ignoring their capacities.^4^ This is balanced by the positive perception of finding help, emphasising the importance of responsive care systems that meet asylum seekers’ legitimate expectations with respectful treatment, clear communication and autonomy.^32^ Concerning the need for calmness and security, other authors also describe how harsh living conditions in reception centres exacerbate stress, while limited psychosocial support contributes to feelings of abandonment and worthlessness.^33^ Policy failures in resource allocation exacerbate challenges in supporting asylum seekers and foster crises in support systems.^33^ The subthemes on the unpredictable future align with Hocking’s study, which highlighted the distress caused by the passive nature of asylum procedures, characterised by waiting, uncertainty and chronic worry.^34^ The evident toll on mental health underscores the need for quicker and more transparent procedures.^17,35^

The third theme, ‘a personal position and individual experience’, was novel compared with earlier research on asylum seekers’ explanatory models. This theme explicitly focused on the personal aspects of individuals’ explanatory models, in comparison with the burdensome past and challenging reality. These aspects manifested themselves in individual characterisations of suffering, surpassing mental limits and affecting self, relationships and help-seeking behaviour. The CFI approach enabled wording of asylum seekers’ own perception of their problems. This ranged from impaired functioning and behaviour to lack of strength to live, difficulties with memories, interpersonal difficulties and bodily problems. They personally experienced distress as a normal reaction to their difficult life circumstances, a recurring perspective in other studies on explanatory models.^2,12^ Our analysis showed how asylum seekers’ suffering exceeded their mental capacities and resilience and how feelings of powerlessness and loss of control affected them. These feelings were repeatedly reported and put in the context of a life that was marked by inactivity, limited agency and a struggle to find distractions, further contributing to a pervasive sense of powerlessness and lack of control.^4,17^ This should urge caregivers to focus on establishing conditions that promote resiliency, to prevent symptoms from evolving into disorders.^36^ Our analysis maps out the impact of suffering on the sense of self of asylum seekers, who often found their self-perception to be shaped by their suffering and felt affected as a human and confronted with a stranger within themselves. The subtheme on the relational aspects of their suffering showed how, despite falling back on themselves, they sought external reassurance, e.g. from fellow asylum seekers or religion. It also emphasised asylum seekers’ profound need for connection with others and for meaningful activity and demonstrated how they experienced their suffering as a social suffering in relationship with others. As shown in other studies, the symptoms are an ‘idiom of distress’ (mode of experiencing and expressing distress) linked to one’s social position and vulnerability, rather than a pathological condition.^4,12^ These findings further resonate with the primary attribution of symptoms to social consequences of resettlement and displacement, such as loneliness, separation from family and living alone, more than migration or trauma-related explanations such as war and fighting.^12^ The preference for social support underscores the significance of social aspects and the importance of a context-sensitive approach.^12,16^ Asylum seekers grapple with their distress, expressing feelings of helplessness and resorting to functioning in ‘survival mode’, often employing concealment as a coping strategy. Earlier research has associated asylum seekers’ helplessness with a lack of autonomy and frustrated ambitions to work, study and rebuild their lives.^34^ Some strive for personal growth amid adversity; this can be related to adversity-activated development^4^ and demonstrates the high resilience of asylum seekers, who often overcome hardship with minimal aid in basic supportive conditions, as well as the need to recognise their individual and collective capacities and strengths.^4^

The role of the CFI in eliciting explanatory models also deserves attention. It helps to understand asylum seekers’ symptoms as a personal ‘idiom of distress’ situated within a socio-relational context in response to a specific situation. The findings resonate with earlier research on cultural formulation that highlighted the importance of a socioculturally contextualised understanding in avoiding misinterpretation and fostering the identification of resources, help-seeking behaviours and coping styles.^21,37^ What stands out in this analysis of the CFI narrative compared with previous studies on asylum seekers’ explanatory models is the centrality of the ‘self’. The central role of the theme ‘a personal position and individual experience’ demonstrated how the CFI can create a potential space for self-determination within the clinical encounter, challenging the medicalisation of distress and embracing uncertainty and opacity.^3^ As the CFI enables open exploration of the personal, familial and social consequences of self-construal, it leaves space for a more nuanced sociocentric or cosmocentric view instead of a Western individualistic worldview. Even without shared mental health assumptions or worldviews, the CFI may enable collaborative exploration of asylum seekers’ perception of causality and problem-solving strategies. Strand suggested that prioritising the CFI’s self-transformative potential, by focusing on the issues that are most urgent, relevant and meaningful to the patient, could even have therapeutic potential beyond solely gathering cultural information.^38^ The emphasis on the ‘self’ in this CFI analysis may be even more therapeutically relevant, as self-narration allows construction of individual and collective identities, which may foster healing through the integration of diverse experiences.^39^ From this perspective, the CFI-guided exploration of explanatory models may serve as a bridge between personal narratives and the eco-social context, facilitating a holistic understanding that may inform person-centred interventions.

Finally, the results of this study offer a possible answer to the ongoing quest to determine how to integrate the CFI’s information into a clinically useful formulation.^40^ The interaction between the themes of our CFI analysis of asylum seekers’ explanatory models of mental illness – past, reality and self – could serve as a basis for case formulation, analogous with Malan’s ‘triangle of person’ used for psychodynamic case formulation.^41^ The structure of our findings offers a dynamic and relational understanding of asylum seekers’ suffering, taking their life history and social context into account. The dynamic nature of this understanding and formulation is important, as it requires clinicians to engage in a ‘not knowing’ stance, recognising the opacity of others’ minds that cannot be overcome, as an essential part of human interrelatedness.^42^ This ‘negative capability’ (being able to tolerate not knowing) can foster clinicians’ reflections on personal biases, including social context, interests, countertransference and power dynamics.^43^ It also allows joint exploration of the personal meaning of suffering in the here and now. In this way, CFI-guided exploration of first-hand explanatory models of mental illness creates room for the deeply personal significance of the present situation, including the asylum seeker’s values and strengths. Engaging with this personal significance presents an opportunity to therapeutically utilise these strengths, despite being stuck in a painful past and quasi-impossible reality.

### Limitations

For the first time, this research on the CFI focused exclusively on asylum seekers with their specific legal and uncertain social context. A major limitation of this study was that the described value of the CFI was based on an intervention without a control group. This prevented us from considering whether the CFI approach yielded a substantially different understanding of explanatory models than treatment as usual. Although the CFI has been examined in ethnically different populations, to date it is unknown whether it is an acceptable, feasible and useful tool for asylum seekers. Consequently, it is unclear whether misunderstanding or restrictive responsiveness, for instance, might have affected the responses. This was among the few studies on the CFI that have used certified interpreters as necessary.^44^ Working with interpreters allowed the participants to express themselves in their native language. Although this may have resulted in translation difficulties, it also ensured the clinical representativeness of our findings. The sample consisted predominantly of males, so the experiences of other genders were not well represented. In addition, although participants were recruited from different reception centres, all interviews were conducted by the main clinician–researcher. A larger study sample with a larger team may strengthen the transferability of the findings. Finally, the high variety among the participating asylum seekers in this study should be noted. More precise information on the participants’ sociodemographic and migration backgrounds might have strengthened our understanding of the broad variance between asylum seekers and the possible impact on their explanatory models.

Several precautions were taken to ensure methodological quality.^22^ The Consolidated Criteria for Reporting Qualitative Research guidelines were used to report on the methods and results of this study.^45^ A protocol paper was previously published, with the aim of ensuring transparency and transferability.^22^ Although formal member checking was not feasible, summary questions were used to ensure an accurate understanding and credibility of our findings. The first author (L.C.) conducted the interviews. He is a White cisgender man of Belgian origin and a resident psychiatrist, psychodynamic psychotherapist and PhD researcher. He was introduced to the participants as a medical doctor–researcher. We are aware that this may have affected their experience and possibly created a sense of an unequal power balance. The potential introduction of personal bias by involvement in participant interviews is acknowledged by the project’s social constructivist stance. The research team comprises all clinical psychiatrists experienced in cultural psychiatry. Commitment to advocating for the mental health of asylum seekers may have influenced their attitudes towards the findings.^22^

### Implications

Understanding asylum seekers’ explanatory models is vital for their inclusion in healthcare services. The CFI offers a culturally sensitive method for exploration and construction of first-hand explanatory models. This study aimed to analyse asylum seekers’ explanatory models of mental illness as elicited by the CFI. The themes ‘burden of the past’ and ‘a disenabling current reality’ highlighted the interplay between pre-, peri- and post-migration experiences and the continuous impact on asylum seekers’ mental health. The theme ‘a personal position and individual experience’ revealed how the CFI enables self-determination in clinical encounters, questioning the medicalisation of distress and embracing uncertainty. This process of self-determination is important to create an opening for interventions that emerge from asylum seekers’ strengths.

Our findings show for the first time how the CFI can be used as a context-sensitive and effective semi-structured approach for eliciting asylum seekers’ explanatory models in a clinical setting. They demonstrate how the CFI fosters the co-creation of a dynamic understanding of asylum seekers’ suffering and emphasise understanding of symptoms as a personal ‘idiom of distress’ situated within a socio-relational context in response to a specific situation. Further research is required to evaluate asylum seekers’ experiences with the CFI and its effects on clinical outcomes.^22^ Investigating implementation strategies to enhance the CFI’s integration into routine clinical practice is an important next step.^46^

## Data Availability

The data that support the findings of this study are available from the corresponding author, L.C., upon reasonable request. The data are not publicly available for reasons of participants’ privacy.
